# Mannose-engineered nanoparticles for site-specific delivery of fluconazole to treat cutaneous leishmaniasis

**DOI:** 10.7717/peerj.21566

**Published:** 2026-07-14

**Authors:** Zahra A. Alamri, Noha T. Zelai, Amaal H. Hassan

**Affiliations:** 1 Department of Biological Sciences, Faculty of Science, King Abdul Aziz University, Jeddah, Makkah, Saudi Arabia; 2 Department of Biology, Faculty of Science, King Khalid University, Abha, Saudi Arabia

**Keywords:** Cutaneous leishmaniasis, Fluconazole, Nanoparticles, Chitosan, Mannose

## Abstract

**Background:**

Cutaneous leishmaniasis (CL) is a skin disease that affects people living in arid and semi-arid regions. The main limitation in current management is that treatment primarily targets the cutaneous lesions, while immune-mediated effects is often not addressed. In addition, there is a need for new treatment with minimized side effects. Fluconazole (FCZ) has been used for treating CL before.

**Methods:**

In the present study, FCZ was entrapped in a chitosan (CS) nanopolymer and conjugated with mannose (MNS) to target amastigotes inside macrophages in CL-infected Balb/c male mice. Parasites were detected using Enzyme-Linked Immunosorbent Assay (ELISA) and Polymerase chain reaction (PCR).

**Results:**

The resulting FCZ CS MNS NPs nanoparticles exhibited small size (71.96 ± 11.4 nm) good stability (31.2 ± 0.173 nm), release over an extended period, and long-term bioavailability. The FCZ CS MNS nanoparticle-treated mice showed negative PCR results and low IgG levels compared to the positive control (*P* < 0.0001). This indicated potent targeting of infected macrophages that affects the parasite before it replicates and exits into the bloodstream. This study provides evidence for the effect of an antileishmanial formulation. Additional toxicity studies should be performed on this formulation before it undergoes clinical trials.

## Introduction

Cutaneous leishmaniasis (CL) is a neglected disease caused by *Leishmania* parasites. These parasites include several species that reside in the macrophages of the dermal reticuloendothelial system. CL has spread in East and Sub-Saharan Africa, the Middle East, the Americas, and South and Central Asia, with more than 700,000 to 1 million reported cases annually (Alvar et al., 2012; WHO, 2023). It is transmitted by female phlebotomine sand flies, which grow in warm weather under low humidity (Saadene & Salhi, 2025). CL is characterized by localised skin lesions and ulcers in both human and mice. The most challenging issue with this disease is that most patients focus on getting the topical symptoms treated. In these cases, parasites persist inside the body even after the ulcer heals (Severino et al., 2022). Therefore, an effective treatment that kills the causative agent is required.

Since conventional medications such as antimonials and amphotericin B are associated with high toxicity and rising parasite resistance (WHO, 2010; Sundar & Chakravarty, 2015), fluconazole (FCZ) was used as an alternative choice in the current study. FCZ is an oral antifungal azole which has a favorable safety profile, tissue penetration and lower cost compared to the standard treatments of CL (Alrajhi et al., 2002). Several studies have confirmed the efficacy of FCZ in CL treatment (Alrajhi et al., 2002; Aburabie et al., 2020). However, some studies have reported treatment failure (Francesconi et al., 2018) or the need for high concentrations of FCZ (Sousa et al., 2011). Thus, there is a need to enhance the effect of FCZ using a novel technique that improves its drug ability (Nahanji et al., 2024; Oliveira et al., 2015).

The use of mannose (MNS) in nanoformulations facilitates the targeting of MNS receptors in *Leishmania*-infected macrophages. These receptors are expressed in macrophages and play major roles in endocytosis and antigen presentation (Cummings, 2022). Therefore, MNS has been widely used in the preparation of antileishmanial drug formulations (Ghosh et al., 2017; Dowari et al., 2022; Barros, Lima & Cordeiro-da Silva, 2015).

Chitosan (CS) is a natural polymer with antimicrobial and antiparasitic activities, low toxicity, biodegradability, and biocompatibility. These properties make CS a good candidate for biomedical applications, including drug delivery (Yadav, Malviya & Kaushik, 2024). Therefore, this study aimed to develop and evaluate an intramuscularly administered fluconazole-loaded chitosan nanoparticle formulation, further functionalized with mannose, to enhance infected macrophage-targeted drug delivery and improve antileishmanial efficacy by increasing intracellular drug uptake and parasite inhibition.

## Materials & Methods

### Mouse groups

Eight groups of healthy male BALB/c mice were used in this study after a 7-day acclimation period. Each group contained nine mice per cage, according to a previous study (Saberi et al., 0000) weighing 25–30 g, purchased from the animal house of The Faculty of Veterinary Medicine, Assiut University. The exclusion criteria included any animal showing health deviations before allocation. All animals were analyzed, as none showed deviation. All the seventy-one mice were provided water and food ad libitum and housed in standard cages on a 12-h light/dark cycle. All interventions were explained in the Experimental Design section. No analgesia was given as CL causes painless ulcers. After the completion of the treatment course, outcomes included assessment of infection burden through quantification of parasite DNA by Polymerase chain reaction (PCR) (*n* = 3) and anti-leishmanial IgG levels by Enzyme-Linked Immunosorbent Assay (ELISA) (*n* = 9 for FCZ groups and *n* = 5 for control, free FCZ vehicle, and blank CS nanoparticles (NPs) and CS MNS NPs groups); only some animals were included in each analysis due to limited blood volume. Blood samples were collected through retroorbital sinus of the mice under anaesthesia of isoflurane in oxygen inhalation. At the end of the study, all surviving animals were euthanized using cervical dislocation. This study was conducted in a laboratory equipped for hosting *Leishmania* researches at the animal house of The Faculty of Veterinary Medicine, Assiut University, in accordance with the ARRIVE guidelines and approved by the Ethical Committee of The Faculty of Veterinary Medicine, Assiut University, Assiut, Egypt, according to The OIE standards for the use of animals in research (Approval No: 0354).

### Parasite

*Leishmania major* was used in this study. To perform animal experiments, *L. major* was injected subcutaneously into each mouse at an infection load of 10^7^ parasites/mL (Sinha et al., 2014).

### Preparation of free FCZ and vehicle

Free FCZ drug was prepared by dissolving 0.1 g FCZ in 100 mL hot distilled water (DH_2_O). Blank vehicle contained hot DH_2_O only.

### Preparation of FCZ-encapsulated CS nanoparticles (FCZ CS NPs)

Chitosan (CS) nanoparticles were prepared as previously described by El Rabey et al. (2019) and Chopra et al. (2014). Briefly, a CS solution (0.5% w/v) was prepared in 1% acetic acid solution containing 0.5% w/v Tween 80. Then, 0.1 g FCZ was dissolved in 100 mL of hot DH_2_O and added dropwise to the CS solution at 15% w/w. Subsequently, tripolyphosphate was added dropwise to the solution under magnetic stirring for 20 min. The mixture was then centrifuged for 30 min with cooling (4 °C) at 15,000 rpm (Hermle Z32 HK, Germany). Blank nanoparticles were obtained by adding a tripolyphosphate aqueous solution to a CS solution.

### Preparation of mannosylated FCZ-encapsulated CS nanoparticles (FCZ CS MNS NPs)

Briefly, two mL MNS solution was prepared by adding 10 mg MNS to water at pH 4 (Pawde et al., 2020). This solution was then added to the FCZ CS NPs mixture and gently stirred for 48 h. MNS molecules underwent a ring-opening reaction due to the acidic environment, and the aldehyde moiety interacted with the free amino group located on the exterior of CS, forming a Schiff’s base (-N=CH-). Uncoated MNS and other impurities were removed by placing the prepared FCZ CS MNS NPs on a dialysis membrane (12 kDa, SERVA, Germany) for 30 min with double-distilled water in the receptor compartment (Vieira et al., 2018; Guo et al., 2014). A blank vehicle was prepared using the same method, but without adding FCZ (Pawde et al., 2020).

### Characterization of FCZ Nanoformulations (Size, shape, and charge density)

To determine the size and shape of the nanoparticles, a high-resolution transmission electron microscope (JEOL JEM-2100, JEOL, Freising, Germany) was operated at an accelerating voltage of 200 kV, and subsequent analyses were performed using SerielEM image analysis software. Samples were prepared by placing a droplet of colloid suspension onto a Formvar carbon-coated, 300-mesh copper grid (Ted Pella) and allowing it to dry by evaporation under ambient conditions (Helal et al., 2022). A Zetasizer ZS90 (Malvern Instruments, Malvern, UK) was used to determine the charge density of the nanoparticles (Helal et al., 2022).

### Loading efficiency

A stock solution (200 µg/mL) was prepared by dissolving 20 mg FCZ in phosphate buffer (pH 7.4) and then adjusting it to a final volume of 100 mL. Solutions with concentrations ranging from 200 g/mL to 10 µg/mL were prepared from this stock and analysed using a UV-Vis spectrophotometer (Cary series UV-Vis- NIR, Australia) adjusted to 260 nm to plot a calibration curve. The amount of encapsulated FCZ was determined from the absorbance of the separated supernatant containing the free drug after centrifugation at 20,000 rpm for 60 min at 4 °C. The amount of free FCZ was estimated based on the absorbance calculated from the standard curve. The loading efficiency was calculated using the following formula (Kelidari et al., 2017):

\begin{eqnarray*}\text{Entrapment efficiency}= \frac{Initial~concentration-Free~concentration}{Initial~concentration} \times 100. \end{eqnarray*}

### FTIR spectroscopy

Samples were analysed using an FTIR-ATR Spectrometer (FT-IR vertex 70 RAM II, Bruker, USA) over 4,000–400 cm^−1^ (Helal et al., 2022).

### *In vitro* release study

To preliminary evaluate the drug release behavior, a suspension containing 1 g FCZ CS NPs was prepared in five mL of PBS (pH 7.4). After placing the suspension in a dialysis bag (MWCO 10:12 KDa), the bag was immersed in 50 mL of PBS solution at 37 °C. The cumulative percentage release of FCZ was calculated using the following equation:

\begin{eqnarray*}\text{Cumulative release}= \frac{Total~volume~of~released~solution+Volume~per~sampling}{Initially~loaded~amount~of~FCZ} \times 100. \end{eqnarray*}

The total volume of the released solution was 55.0 mL and the volume per sampling was three mL (Helal et al., 2022).

### Pharmacokinetic evaluation

The FCZ levels in mouse plasma were tracked using the method described by Santos et al. (2010). To prepare the standard curve, blank plasma samples were spiked with serial concentrations of FCZ from 100 to 0.2 µg/mL and 50 µg/mL of carbamazepine dissolved in methanol was added as an internal standard (IS). The IS was evaporated to obtain dry carbamazepine films in each tube. Blood samples were collected from each group (*n* = 3; samples were combined to obtain a higher yield) at 0, 1, 8, 24, 48, and 72 h. These samples were centrifuged at 3,000 rpm for 30 min, and the plasma was separated and stored at −80 °C for further analysis. The pH of the collected plasma samples (50 µL) was adjusted to alkalinity by adding 50 µL of 1.25 M NaOH and vortexing the mixture for 10 s. Subsequently, three mL dichloromethane was added and shaken for 1 min, and FCZ and its IS were transferred to the organic solvent. The mixture was then centrifuged at 3,000 rpm at 4 °C for 30 min. The organic solvent at the bottom of the tube was evaporated in a 37 °C water bath, to extract FCZ and its IS. The extracted drugs were mixed with the mobile phase and analysed.

The standard and test samples were injected into a high-performance liquid chromatograph (HPLC) system (LC-10AVP, Shimadzu, Kyoto, Japan) with a C18 (150 × 6.0 mm) column, using a mobile phase of water and acetonitrile (60:40 v/v). HPLC conditions were adjusted to maintain a flow rate of 0.5 mL/min, injection volume of 5 µL, and wavelength of 210 nm for 15 min at room temperature.

### Experimental design

An a priori protocol was prepared before data collection; the study was registered as a PhD proposal for the first author Z.A A. at Zoology Program, Department of Biological Science, King Abdulaziz University. Male mice were divided into eight groups of nine mice; seven groups were infected with the parasite and randomly distributed among seven cages. The cages were positioned at fixed locations in the lab and labeled 1-8; treatments were applied in that order throughout the experiment. The negative control group included mice that were not infected with *Leishmania* or treated, whereas the positive control group included mice that were infected but not treated. The free FSZ group included mice that were infected with *Leishmania* and then treated with 5 mg/kg FCZ for 15 d (Riezk, 2020). The free FCZ vehicle group included mice that were infected and then treated with DH_2_O (the solvent of FCZ). The nanoparticle group included mice that were infected and then treated with 5 mg/kg of FCZ CS NPs for 15 d, while the nanoparticle vehicle group included mice that were infected with *Leishmania* and then treated with a blank CS nanocarrier. The mannosylated nanoparticle group included mice that were infected with *Leishmania* and then treated with 5 mg/kg FCZ CS and MNS-conjugated nanoparticles (FCZ CS MNS NPs) for 15 d, and the mannosylated nanoparticle vehicle group included those infected with *Leishmania* and then treated with blank mannose-conjugated nanoparticles (CS MNS NPs). This study used investigator blinding; group allocation was concealed until completion of the primary analysis.

### Parasite detection

The study endpoint was completion of the 15-day treatment course. The persistence of *Leishmania* after treatment was assessed by PCR and ELISA. One day after treatment, blood samples were collected from the retroorbital sinus of the mice in EDTA tubes for PCR analysis or in anticoagulant tubes for ELISA.

### Molecular detection

DNA extraction: DNA was extracted from three samples from each group, using QIAamp DNA Blood Kits (Qiagen, Hilden, Germany) following the manufacturer’s protocol. PCR amplification: PCR was performed using the CFX96 Real-Time PCR Detection System (Bio-Rad Laboratories, Hercules, CA, USA) according to the method described by Van der Meide et al. (2008). A 171-bp fragment of the 18S ribosomal RNA (18S rRNA) gene of *Leishmania* spp. was targeted for each sample. The forward and reverse primers for detecting *Leishmania* DNA are 5′-CCAAAGTGTGGAGATCGAAG-3′ and 5′-GGCCGGTAAAGGCCGAATAG-3′, respectively. Extracted DNA was treated to obtain a final volume of 20 µL by mixing 1 µL of 10 µM primers with 0.5 µL of 10 µM Le 18S probe, 2 µL of gDNA, and 5.5 µL of nuclease-free water with 10 µL of Taq polymerase (Bio-Rad Laboratories). The thermocycler was optimised to perform initial denaturation of 95 °C for 2 min and 45 cycles of denaturation at 95 °C for 15 s, and 60 °C for 15 s. Cycle threshold (Ct) values lower than 40 were considered to indicate the presence of parasitic molecules (positive), while Ct values greater than 40, were interpreted as negative for parasite presence. ImageQuant (Cytive, United States) for Windows was used to quantify PCR data.

PCR results were analyzed qualitatively as positive or negative based on Ct thresholds due to the exploratory nature of the study and the small sample size (*n* = 3). Although quantitative qPCR is commonly used to estimate parasite load in leishmaniasis, reliable quantification requires larger cohorts and validated standard curves. Therefore, the present findings should be interpreted as indicative of parasite detection rather than definitive parasite clearance, and further studies using quantitative approaches are warranted.

### Sero-detection enzyme-linked immunosorbent assay (ELISA)

Mouse *Leishmania* spp. isolates were qualitatively analysed using an antibody (antiLS) ELISA Kit (Cat No. MBS9906134). The ELISA method described by Sarkari et al. (2014) was used, with minor modifications. Serum samples from each group (*n* = 9 for FCZ groups and *n* = 5 for control and vehicle groups) were placed in a 96-well flat-bottom plate to detect anti-*Leishmania* antibodies. A total of 50 µL containing 10 µL of the sample and 40 µL of the sample diluent were added to the sample wells. Then, 100 µL of horseradish peroxidase-conjugate reagent were added and the plate was covered with an adhesive strip and incubated at 37 °C for 1 h. Next, 50 µL Chromogen Solution A followed by 50 µL Chromogen Solution B were added to each well with gentle mixing and the plate was incubated at 37 °C for 15 min. Stop solution (50 µL) was added to each well. The absorbance test results were read 15 min after adding the stop solution using an ELISA Plate Reader (Bio-Rad Laboratories) at 450 nm.

### Statistical analysis

All data related to nanoparticle size, zeta potentials, antibody levels, and ct values were presented as the mean ± S.E.M, with the corresponding 95% confidence intervals for all formulations. One-way analysis of variance was used to determine the significant differences among the tested groups. Tukey’s *post-hoc* test was conducted to determine the differences between the two groups. A *p*-value < 0.05 was considered statistically significant, while *p*-values ≥ 0.05 were considered not significant. Drug release profiles and pharmacokinetic data were obtained from a combined single sample. Thus, correlation coefficients (r) and two-tailed *p*-values were used to determine the correlation between drug release in all groups. The statistical significance of the ELISA results and PCR cycle threshold values across all experimental groups was also assessed using ANOVA, followed by Tukey’s *post-hoc* test for multiple comparisons.

## Results

To overcome the limitations associated with conventional antileishmanial drugs, a mannosylated chitosan nanoparticles delivery system was developed. The following results first confirm the physicochemical integrity of the prepared formulations and then demonstrate its influence on drug encapsulation, release behavior, and therapeutical effects.

### Characterization of FCZ nanoparticles

**Figure 1:**
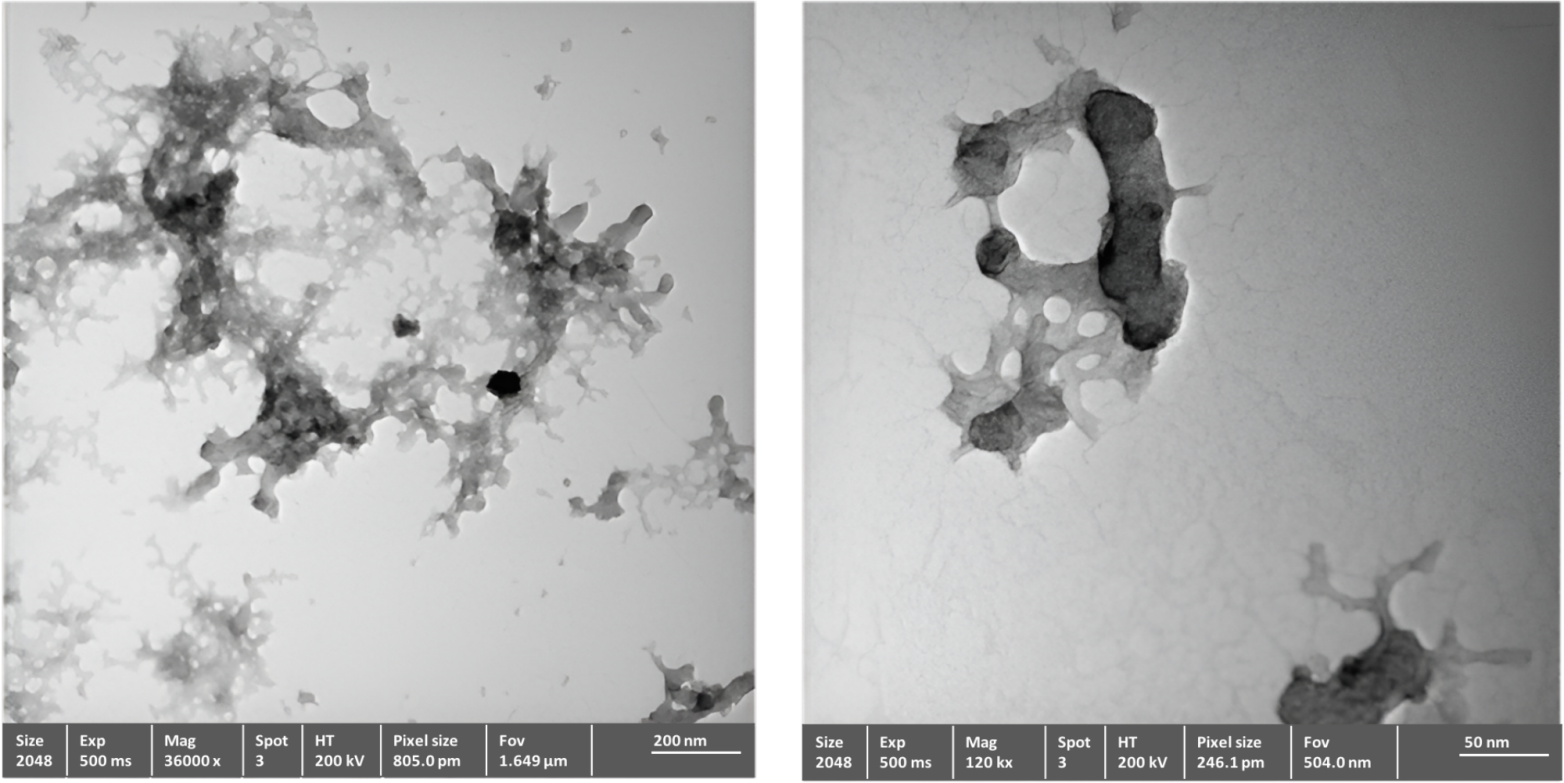
SEM image illustrating the spherical shape of nanoparticles.

**Figure 2:**
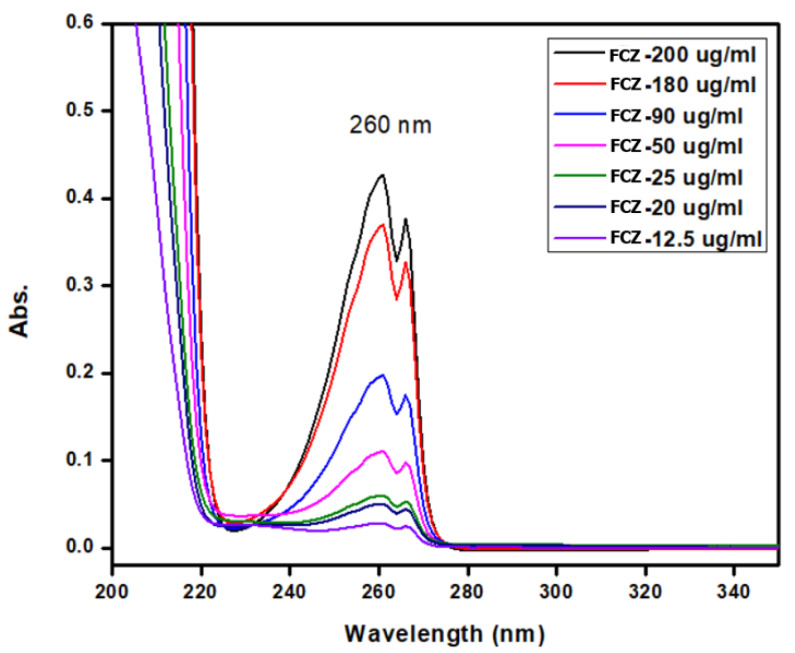
Absorption spectra at different FCZ concentrations.

**Figure 3:**
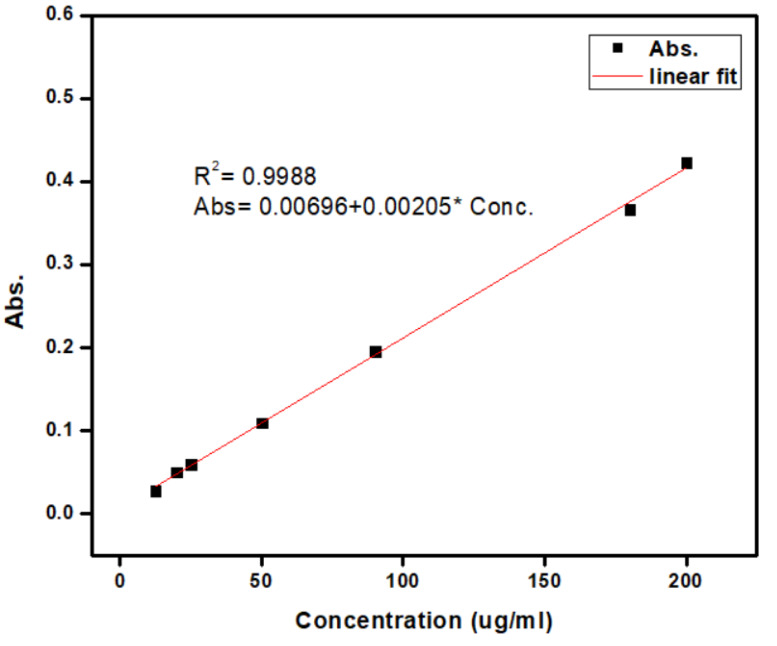
Calibration curve for FCZ.

**Figure 4:**
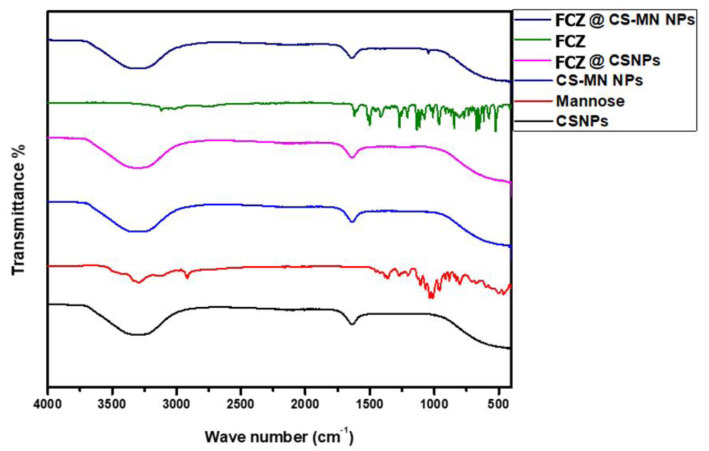
FTIR spectra of prepared materials.

In this study, two FCZ nanoparticle formulations were prepared, namely FCZ CS NPs and FCZ CS MNS NPs, and two FCZ-free formulations, similar to the previous formulations, served as vehicles. The resulting formulations were spherical (Fig. 1); their average size is shown in Table 1. Minimal-sized particles were achieved in the blank CS NPs formulation which was significantly smaller than that of FCZ CS MNS NPs (*P* = 0.003). Based on the zeta potentials, the mannosylated groups FCZ CS MNS NPs (31.2 ± 0.173) and blank CS MNS NPs (31.6 ± 0.95) have similar particle charge (*P* = 0.998), although they differ from the other groups (*P* < 0.0001). FCZ was detected by measuring absorbance at a wavelength of 260 nm (Fig. 2). After drawing the calibration curve (Fig. 3), the loading efficacy was calculated as 86 ± 2.4%. In the CS NPs (black spectrum; Fig. 4), the broad peak at 3,360 cm^−1^ was assigned to the stretching vibration mode of N–H, overlapping with the O–H stretching vibration mode. The NH bending vibration peak at 1,580 cm^−1^ shifts to 1,635 cm^−1^ (Hasanin et al., 2018).

The vibrations of the various functional groups in FCZ are illustrated in the green spectrum in Fig. 4. The molecular bands at 1,618 cm^−1^ and 1,513 cm^−1^ were assigned to the C=C stretch of the aromatic ring, whereas the bands at 1,500 cm^−1^, 1,463 cm^−1^, 1,451 cm^−1^, and 1,417 cm^−1^ were attributed to the triazole ring stretching. Peaks at 1,270 cm^−1^ were assigned to the C–F stretch, 1,134 cm^−1^ to the triazole ring, 1,025 cm^−1^ to the C–H aromatic ring, and 966 cm^−1^ and 845 cm^−1^ were attributed to the C–H triazole ring (Teleginski et al., 2015).

In the spectrum of D-mannose (red spectrum; Fig. 4), the peak around 3,420 cm^−1^ was assigned to the vibration of free -OH groups at the asymmetrical carbon of the mannose sugar. The peaks at 3,292 cm^−1^ and 3,127 cm^−1^ corresponded to the C–H stretching groups of the aromatic protective group from the sugar skeleton, while bands at 2,978–2,926 cm^−1^ denoted the CH2 group from the furanosic sugar rings. Peaks at 1,380 cm^−1^ and 1,035 cm^−1^ are attributed to the C–O–C asymmetric frequencies.

**Table 1:** Average size and zeta potentials of the nanoparticle formulations.

**Formulation**	**Average size ± S.E.M (nm)**	Zeta potential (mV)
FCZ CS NPs	52.8 ± 7.7	24.7 ± 0.87
FCZ CS MNS NPs	71.96 ± 11.4	31.2 ± 0.173
Blank CS NPs	27.455 ± 6.7	40.7 ± 1.52
Blank CS MNS NPs	41.2 ± 2.5	31.6 ± 0.95

**Notes.**

FCZ Fluconazole CS hitosan NPs Nanoparticles MNS Mannose

For the FCZ CS MNS NPs (dark blue spectrum), the new peak at 1,045 cm^−1^ was assigned to the triazole ring stretch of FCZ, indicating conjugation between the CS MNS NPs and FCZ (Pană et al., 2013).

In the FTIR spectrum of the CS MNS conjugate (light blue spectrum), two peculiar peaks were observed at 1,560 cm^−1^ and 1,456 cm^−1^ indicating N–H bending of the secondary amine and C=N stretch, respectively.

### *In vitro* release study

**Figure 5:**
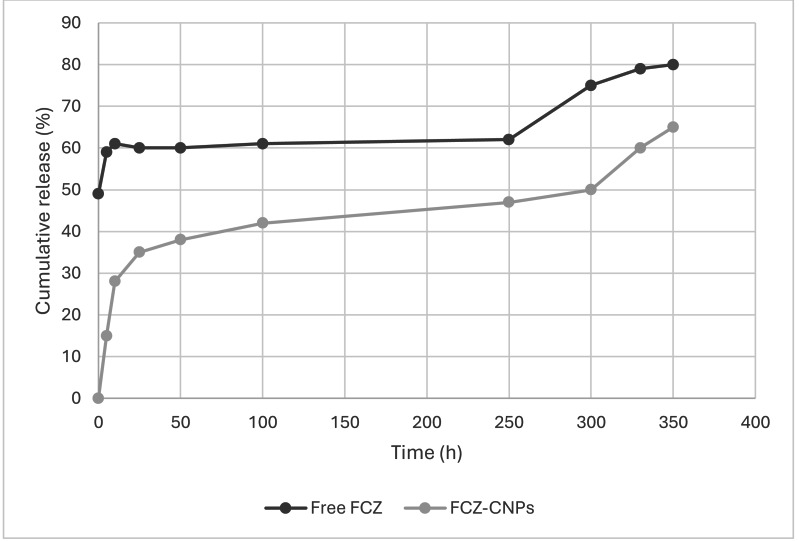
Representative *In vitro* release behavior of free FCZ and FCZ CS NPs in PBS (pH 7.4).

The preliminary *in vitro* behavior of FCZ (Fig. 5) showed that both free and nanoparticle formulations followed a three-phase release. The release began with bursts of 61% and 38% in the free and nanoparticle formulations, respectively. Then, it was almost stable until 250 h. Finally, a quick release was observed which peaked at 80% and 65% in free and nanoparticle formulations, respectively, within 14.5 d. Pearson correlation revealed that the two curves corresponded to each other closely over time (*r* ≈ 0.89; *P* < 0.001).

### Pharmacokinetic evaluation

**Figure 6:**
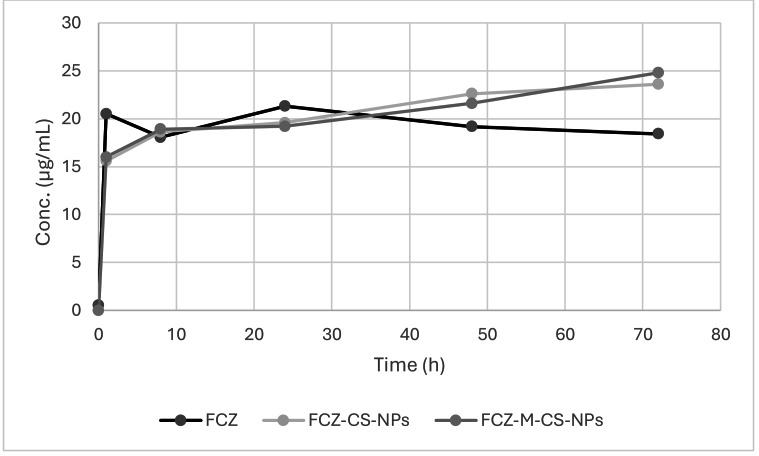
Representative pharmacokinetics profile of free FCZ and FCZ CS NPs in PBS (pH 7.4) (*n* = 3 combined samples).

Blood plasma was extracted from mice that received the treatment for 3 d. The pharmacokinetic study (Fig. 6) showed a quicker release of free FCZ than of FCZ from the nanoparticle formulations. In addition, similar release profiles (*r* ≈ 0.99) displaying accumulation were observed in both FCZ CS NPs and FCZ CS MNS NPs. However, the correlation among the three groups was strong and significant (*r* = 0.9, *P* < 0.001).

### ELISA

**Figure 7:**
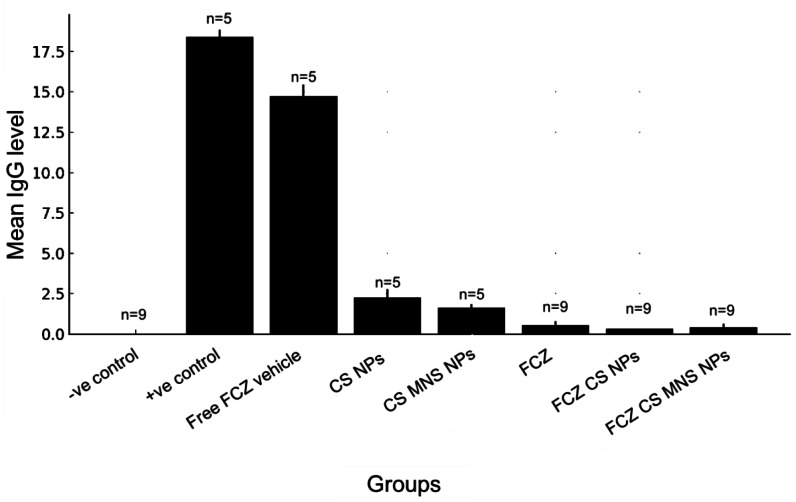
Antibody levels after treatment evaluated by ELISA (*n* = 9 for FCZ groups and *n* = 5 for control and vehicle groups).

Serum levels of *Leishmania* antibodies were evaluated using ELISA (Fig. 7). The mouse serum was extracted and collected at the end of the study. One-way ANOVA showed a significant difference among all groups (*P* < 0.0001). The antibody levels in both the positive control and distilled water-treated groups were significantly higher than those in the other groups (*P* < 0.0001). Antibody levels in the FCZ nanoparticle-treated groups were similar to those in the free FCZ group (*P* > 0.05). Although mice treated with the blank nanoparticle formulations CS NPs and CS MNS NPs showed higher antibody levels than the FCZ nanoparticle-treated mice, this difference was not significant (*P* > 0.05).

### PCR

The PCR results are summarized in Table 2. Three mice from each group were tested for PCR positivity. ANOVA revealed a significant difference among the eight groups (*P* < 0.0001). Although the free FCZ- and CS-MNS NPs-treated groups tested positive for the presence of *Leishmania* as their Ct values were less than 40, this result was not significantly different compared to the negative control group (*P* = 0.1503 and *P* = 0.0690). In addition, there was a significant difference between the positive control and free FCZ groups (*P* = 0.0124), although both were positive for *Leishmania*. The FCZ CS MNS NPs group showed higher Ct values than both the FCZ CS NPs and free FCZ groups, although these differences were not significant (*P* = 0.9953 and *P* = 0.8804).

**Table 2:** Parasite detection using PCR (*n* = 3).

GROUPS	Mean Ct value ± S.E.M.	*Leishmania* detection result
Negative control	44.15847 ± 2.57	Negative
Positive control	31.8608 ± 0.79	Positive
Free FCZ vehicle	31.94347 ± 1	Positive
FCZ	39.1804 ± 1.03	Positive
CS NPs	36.56433 ± 0.55	Positive
FCZ CS NPs	40.23207 ± 0.76	Negative
CS MNS NPs	38.4096 ± 0.63	Positive
FCZ CS MNS NPs	41.4792 ± 1.30	Negative

## Discussion

The current study combined the beneficial effects of FCZ with specific targeting using MNS, in addition to the use of CS, an antiparasitic agent, as a polymer. No expected or unexpected adverse events were observed during the 15-day treatment period. The advantages of nanotechnology were exploited to prepare formulations with small average sizes. The small particle size achieved in this study is an advantageous characteristic that increases cellular uptake (Behzadi et al., 2017). MNS decoration increases the average size from 52.8 ± 7.7 nm to 71.96 ± 11.4 nm. This may be attributed to the increased surface area and hydrodynamic diameter after MNS addition. Previous studies have also found that MNS conjugation in CS nanoparticles similarly increased particle size (from 225.9 ±12.1 nm to 256 ± 12.1 nm (Peng et al., 2015)) and greatly increased particle size in solid lipid nanoparticles (from 160 ± 9 to 252 ± 2 nm (Vieira et al., 2018)).

The addition of MNS resulted in the formation of more stable formulation, as evidenced by the higher positive zeta potential (31.2 ± 0.173 mV) compared to that of non-mannosylated nanoparticles (24.7 ± 0.87 mV). The more cationic surface of the MNS-conjugated group may be due to the acidic environment which protonates the amine group of CS and increases the positive charge of the formulation (Ramanery, Mansur & Mansur, 2013). In addition, these strongly positively charged particles have a higher force of action and a more toxic effect on infected cells (Shao et al., 2015).

Peak vibrations in CS NPs (Hasanin et al., 2018), FCZ (Teleginski et al., 2015), and D-mannose (Pană et al., 2013) were identified and the synthesis of nanoparticles with conjugated MNS were confirmed using FTIR analysis. FCZ engagement was evidenced by the presence of the new band of triazole ring stretching at 1,045 cm^−^^1^ in FCZ CS MNS NPs. In addition, the formation of Schiff’s base (R-CH = N-R bond; peaks at 1,560 cm^−^^1^ and 1,456 cm^−^^1^) by the ring-opening reaction of MNS followed by the reaction of the aldehyde group with the amino group of CS confirmed the formation of MNS-conjugated CS in FCZ CS MNS NPs.

The triphasic *in vitro* release profile showed that free FCZ had a high initial burst rate and a high release percentage of 80% within 15 days. However, the FCZ CS NPs showed a drug release of 65% within the same period. This indicated that the encapsulation of FCZ prevented drug leakage and resulted in a more controlled release than that of the free drug. A similarly higher release was also observed by Nahanji et al. in free FCZ compared to the nano-emulsion formulation (Nahanji et al., 2024).

Pharmacokinetic evaluations also confirmed the slower release of FCZ CS NPs, indicating that this formulation has a higher bioavailability and can be maintained in the plasma at high concentrations over an extended period. Notably, the addition of MNS did not alter the surface of FCZ CS NPs (*r* ≈ 0.99) indicating that MNS is only an external decoration, which is why it was not examined in vitro. Accordingly, *in vitro* and *in vivo* studies demonstrated the sustained release of FCZ CS NPs.

ELISA and PCR were used to assess the effectiveness of the drug formulations. ELISA results showed that free FCZ, FCZ CS NPs, and FCZ CS MNS NPs had lower antibody levels than the positive control group and the group treated only with distilled water. This may indicate that treatment of CL with these formulations eradicates the majority of free parasites before eliciting the production of antibodies. Surprisingly, FCZ-free nanoparticle formulations (CS NPs and CS MNS NPs) showed progressively reduced antibodies, confirming the role of CS as an antiparasitic enhancer in our formulations (Yadav, Malviya & Kaushik, 2024).

PCR is considered an accurate method for confirming the persistence of *Leishmania* in the blood of infected mice. The present results confirmed the efficacy of FCZ CS MNS NPs and FCZ CS NPs in killing the parasite, as evidenced by the high Ct values compared to the positive control group.

## Conclusions

This study provides multiple methods to confirm that CS MNS NPs can be used with FCZ to treat CL. Particle size and zeta potentials showed that the formulation had the favourable physiochemical characteristics of stability and small particle size. *In vitro* and *in vivo* studies suggest improved bioavailability and a prolonged release profile of the nanoparticle formulation compared with the free drug; however, due to the absence of variability measures and limited statistical power, these findings should be interpreted as preliminary. ELISA and PCR showed strong evidence for parasite eradication from blood plasma. This evidence proves that FCZ CS MNS NPs are great candidates for further clinical studies on CL treatment. However, this study has some limitations, including the use of a single sex, single strain, low PCR sample size, lack of replicates in the drug release and pharmacokinetic results and a short (15-day) follow up.

##  Supplemental Information

10.7717/peerj.21566/supp-1 Supplemental Information 1 Raw data

10.7717/peerj.21566/supp-2 Supplemental Information 2 The ARRIVE guidelines 2.0: author checklist

## Additional Information and Declarations

### Data Availability

The following information was supplied regarding data availability: The raw measurements are available in the Supplemental File.
